# The antagonistic pleiotropy of insulin‐like growth factor 1

**DOI:** 10.1111/acel.13443

**Published:** 2021-08-07

**Authors:** William B. Zhang, Kenny Ye, Nir Barzilai, Sofiya Milman

**Affiliations:** ^1^ Department of Medicine Albert Einstein College of Medicine Bronx New York USA; ^2^ Department of Genetics Albert Einstein College of Medicine Bronx New York USA; ^3^ Department of Pathology University of Chicago Chicago Illinois USA; ^4^ Department of Epidemiology and Population Health Albert Einstein College of Medicine Bronx New York USA; ^5^ Department of Systems and Computational Biology Albert Einstein College of Medicine Bronx New York USA

**Keywords:** clinical outcomes, evolution, human aging, IGF

## Abstract

While insulin‐like growth factor‐1 (IGF‐1) is a well‐established modulator of aging and longevity in model organisms, its role in humans has been controversial. In this study, we used the UK Biobank (*n* = 440,185) to resolve previous ambiguities in the relationship between serum IGF‐1 levels and clinical disease. We examined prospective associations of serum IGF‐1 with mortality, dementia, vascular disease, diabetes, osteoporosis, and cancer, finding two generalized patterns: First, IGF‐1 interacts with age to modify risk in a manner consistent with antagonistic pleiotropy; younger individuals with high IGF‐1 are protected from disease, while older individuals with high IGF‐1 are at increased risk for incident disease or death. Second, the association between IGF‐1 and risk is generally U‐shaped, indicating that both high and low levels of IGF‐1 may be detrimental. With the exception of a more uniformly positive relationship between IGF‐1 and cancer, these effects were remarkably consistent across a wide range of conditions, providing evidence for a unifying pathway that determines risk for most age‐associated diseases. These data suggest that IGF‐1 signaling could be harmful in older adults, who may actually benefit from the attenuation of biological growth pathways.

## Graphical abstract

Using a large dataset from the UK Biobank, we show that serum IGF‐1 is associated with risk for mortality and age‐associated morbidity in a manner consistent with antagonistic pleiotropy. While the risk associated with IGF‐1 is U‐shaped at all ages studied, the risk associated with high IGF‐1 increases with age, and the risk associated with low IGF‐1 decreases with age, suggesting that higher serum IGF‐1 may be beneficial in youth but harmful in older age.

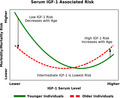

## 1 INTRODUCTION

### 1.1 Aging and disease

Aging is a complex degenerative process that fundamentally modifies risk for disease across all human organ systems (Kennedy et al., 2014), which mainly occurs post‐growth and reproduction. It is the predominant risk factor for numerous debilitating and life‐threatening conditions that afflict older adults, including cardiovascular disease, cancer, and neurodegeneration (Niccoli & Partridge, 2012). To date, a majority of research has focused on the pathogenesis and mechanisms of these individual diseases of aging. However, with the discovery of biological “hallmarks of aging,” fundamental processes which are associated with aging and contribute to its regulation (López‐Otín et al., 2013), there has been increased interest in understanding and targeting the biology of aging itself. Several of the hallmarks of aging, including genomic instability, epigenetic changes, loss of proteostasis, and altered nutrient sensing, contribute to the aging process via accumulation of disruptions; if left uncorrected, these changes eventually perturb cellular homeostasis. Thus, persistent investment in processes that favor growth over repair may result in accelerated accumulation of hallmarks that are associated with aging. This concept mirrors the evolutionary theory of antagonistic pleiotropy, which suggests that aging may emerge from favoring certain processes beneficial in youth but harmful in older age (Williams, 1957). The disposable soma theory, a specific refinement of this idea, argues that the key process involved is energy investment (Kirkwood, 1977): A focus on growth and reproduction might lead to underinvestment in repair and maintenance.

### 1.2 The role of IGF‐1 in aging

As a key mediator of the growth hormone pathway, insulin‐like growth factor 1 (IGF‐1) plays a crucial role in growth and energy investment. It has been shown to modulate aging in a wide variety of model organisms, including the nematode *C. elegans* (Kenyon et al., 1993), fruit flies (Broughton et al., 2005), and rodents (Brown‐Borg et al., 1996). In genetically modified knock‐down and knock‐out models, attenuated growth hormone (GH)/IGF‐1 signaling generally improves the health of mice, delaying age‐associated pathologies such as sarcopenia, immunosenescence, and cancer (Ikeno et al., 2009; Selman et al., 2008; Spadaro et al., 2016). However, sufficient IGF‐1 is also required for normal development and function, particularly in the central nervous system, where it promotes astrocyte activity (Logan et al., 2018) and vascularization (Lopez‐Lopez et al., 2004), and protects against some forms of neurocognitive decline (Farias Quipildor et al., 2019; Gubbi et al., 2018). Given the complex physiologic role of IGF‐1, it is not surprising that human studies of circulating IGF‐1 in aging and age‐associated disease have produced inconsistent results.

In humans, acromegaly and Laron dwarfism provide informative examples of extreme excess or insufficiency of IGF‐1, respectively. In acromegaly, excess growth hormone and resultant excess IGF‐1 cause a variety of detrimental effects, including hypertension, diabetes, cardiac dysfunction, and respiratory disorders (Colao et al., 2004). Laron dwarves, who have attenuated IGF‐1 signaling due to a defective GH receptor, have been shown to have lower rates of cancer, stroke, and diabetes (Guevara‐Aguirre et al., 2011), but may also suffer from obesity, auditory defects, and cognitive deficits (Laron & Kauli, 2016). Taken together, these examples provide some evidence that extremely high IGF‐1 may be detrimental, while extremely low IGF‐1 is associated with a mix of benefits and detriments.

By contrast, epidemiological studies of the naturally occurring variation in circulating IGF‐1 have shown null or mixed results (Milman et al., 2016). Some studies, including those from our longevity cohorts, have shown a positive association between high IGF‐1 and adverse outcomes (Milman et al., 2014; Spoel et al., 2015; Zhang et al., 2020). Others, often from somewhat younger populations, have shown the opposite: higher IGF‐1 being associated with protection from disease and death (Bourron et al., 2015; Friedrich et al., 2009). Perhaps most interestingly, a few studies have also reported a more complex U‐shaped relationship between IGF‐1 and all‐cause (Andreassen et al., 2009), cardiovascular (Burgers et al., 2011), and cancer mortality (Svensson et al., 2012), in which individuals with the highest and lowest levels of IGF‐1 tend to have worse outcomes.

The inconsistencies in previous epidemiological studies of IGF‐1 may be due to insufficient sample size and variations in study population characteristics such as baseline age. To overcome these limitations, we take advantage of the scale of the UK Biobank to probe interactions between age and serum IGF‐1 explicitly, and to more definitively understand the relationship between IGF‐1 and diseases associated with aging.

## 2 RESULTS

### 2.1 Characteristics of the study cohort

**FIGURE 1:**
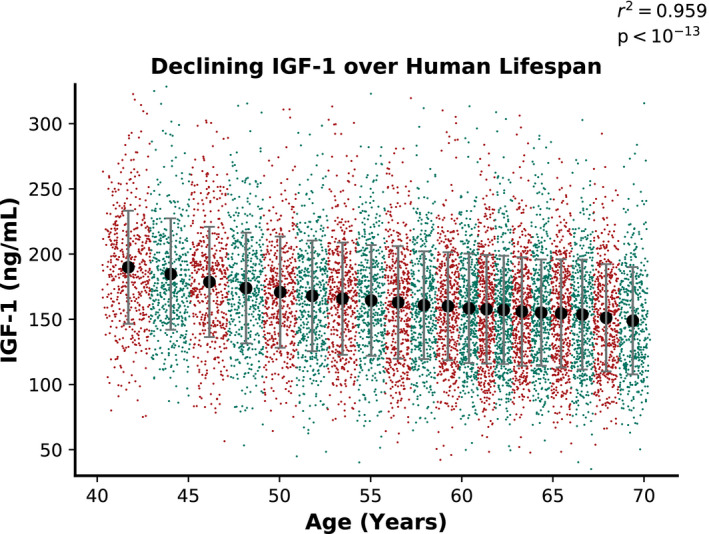
Serum IGF‐1 levels decline with age. Each large dot represents the mean age and serum IGF‐1 level of an age ventile (1/20th of the overall cohort studied, stratified by baseline age). Smaller colored dots indicate 500 randomly selected individuals from each group. The error bars here indicate standard deviations of serum IGF‐1 level within each age group. The *r* ^2^ and *p*‐value reported here are for a linear fit to the group averages (large dots)

This study included 440,185 individuals of European ancestry (54.3% female), who were followed for over 10 years for morbidity and mortality. The baseline characteristics and incident clinical events of the participants are summarized in Table 1. As seen in Figure 1, mean serum IGF‐1 levels decline with age ($p<10^{‐13}$). However, the relatively high variability of IGF‐1 at any given age results in a considerable overlap in IGF‐1 ranges, even between the youngest 5% and the oldest 5% of the cohort. The distribution of IGF‐1 is also similar for men (Figure S1) and women (Figure S2) across ages.

**TABLE 1:** Population characteristics, baseline disease prevalence, and incident morbidity and mortality

	All	Male	Female
*n*	440,185 (100.0%)	201,258 (45.7%)	238,927 (54.3%)
Deaths	18,027 (4.1%)	10,887 (5.4%)	7140 (3.0%)
Baseline Dementia	199 (<0.05%)	108 (0.1%)	91 (<0.05%)
Baseline Vascular Disease	15,886 (3.6%)	11,286 (5.6%)	4600 (1.9%)
Baseline Osteoporosis	2462 (0.6%)	730 (0.4%)	1732 (0.7%)
Baseline Diabetes	21,336 (4.8%)	13,566 (6.7%)	7770 (3.3%)
Baseline Cancer	23,681 (5.4%)	8771 (4.4%)	14,910 (6.2%)
Incident Dementia	2168 (0.5%)	1200 (0.6%)	968 (0.4%)
Incident Vascular Disease	12,002 (2.7%)	7916 (3.9%)	4086 (1.7%)
Incident Osteoporosis	7130 (1.6%)	1162 (0.6%)	5968 (2.5%)
Incident Diabetes	9597 (2.2%)	5531 (2.7%)	4066 (1.7%)
Incident Cancer	26,176 (5.9%)	13,643 (6.8%)	12,533 (5.2%)
Mortality Follow‐up (years)	11.0 ± 1.5	10.9 ± 1.7	11.1 ± 1.4
Morbidity Follow‐up (years)	10.5 ± 1.7	10.5 ± 1.8	10.6 ± 1.5
Age (years)	57.3 ± 8.0	57.5 ± 8.1	57.1 ± 7.9
Age Quartile 1	37.4–50.7	37.4–50.7	39.7–50.5
Age Quartile 2	50.5–58.7	50.7–58.7	50.5–58.0
Age Quartile 3	58.0–64.1	58.7–64.1	58.0–63.4
Age Quartile 4	63.4–73.7	64.1–73.7	63.4–71.1
IGF‐1 (ng/ml)	163.6 ± 43.4	167.7 ± 42.4	160.2 ± 44.0
IGF‐1 Quintile 1	11.1–132.8	14.6–132.8	11.1–122.5
IGF‐1 Quintile 2	122.6–156.7	132.8–156.7	122.6–147.4
IGF‐1 Quintile 3	147.5–176.3	156.7–176.3	147.5–169.3
IGF‐1 Quintile 4	169.3–199.4	176.3–199.4	169.3–194.6
IGF‐1 Quintile 5	194.6–969.6	199.4–969.6	194.6–956.9

### 2.2 IGF‐1‐associated hazard is U‐shaped

To examine the relationship between IGF‐1 and clinical disease, we divided the overall cohort into seven groups based on serum IGF‐1 levels. Relative hazards of a clinical event occurring for groups 1–3 and 5–7 were assessed relative to the middle group, group 4.

**FIGURE 2:**
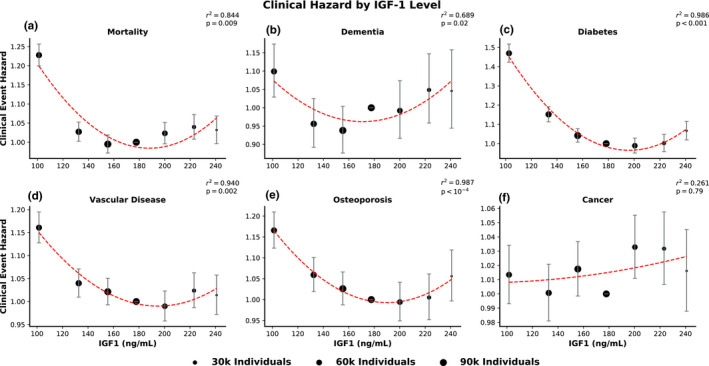
Clinical hazard is increased for both high and low IGF‐1 levels, but not for cancer. Each dot represents the hazard for incident disease or mortality of a subcohort of individuals grouped by IGF‐1 levels, *relative to* the central group, whose hazard is set at 1 by convention. The area of each dot is proportional to the number of individuals it represents, and error bars indicate 95% confidence intervals for hazard ratios. Reported *p*‐values are for an *F*‐test for a quadratic fit to the hazard data, excluding the linear fit component (additional details are available in the methods section). The clinical events evaluated were (a) mortality, (b) dementia, (c) diabetes, (d) vascular disease, (e) osteoporosis, and (f) cancer

We found a strong concurrence between the results for mortality, dementia, diabetes, vascular disease, and osteoporosis. For all five of these clinical events, the relationship between IGF‐1 serum levels and hazard was U‐shaped; elevated hazards occurred at both high and low IGF‐1 levels (Figure 2). A quadratic term significantly improved the fit over a linear model for most outcomes (dementia p=0.001; diabetes p<0.001; vascular disease p<0.001; osteoporosis $p<10^{‐5}$; mortality p=0.001). Notably, a quadratic model did not significantly improve the fit for cancer hazard (p=0.09). When controlled for BMI, the U‐shaped effect remained significant for all conditions, with the exception of cancer, for which it remained non‐significant (Figure S3).

To explore sex‐specific effects, we conducted a stratified analysis for men and women (Figures S4 and S5). While minor differences in the shape of the fit existed, the U‐shape effect tended to apply similarly to both men (dementia p=0.03; diabetes $p<10^{‐4}$; vascular disease p=0.001; osteoporosis p=0.007; mortality p=0.002) and women (dementia p=0.001; diabetes p=0.004; vascular disease p<0.001; osteoporosis $p<10^{‐4}$; mortality p=0.001). Cancer was again the exceptional case without a significant U‐shape effect in either sex (men p=0.0505; women p=0.13).

### 2.3 IGF‐1 interacts with age to modify hazard for death and disease

To determine whether the risk associated with IGF‐1 is uniform across all ages, we stratified the overall cohort into seven groups based on age and assessed the hazard associated with IGF‐1 within each age group, adjusted for residual age differences and sex.

**FIGURE 3:**
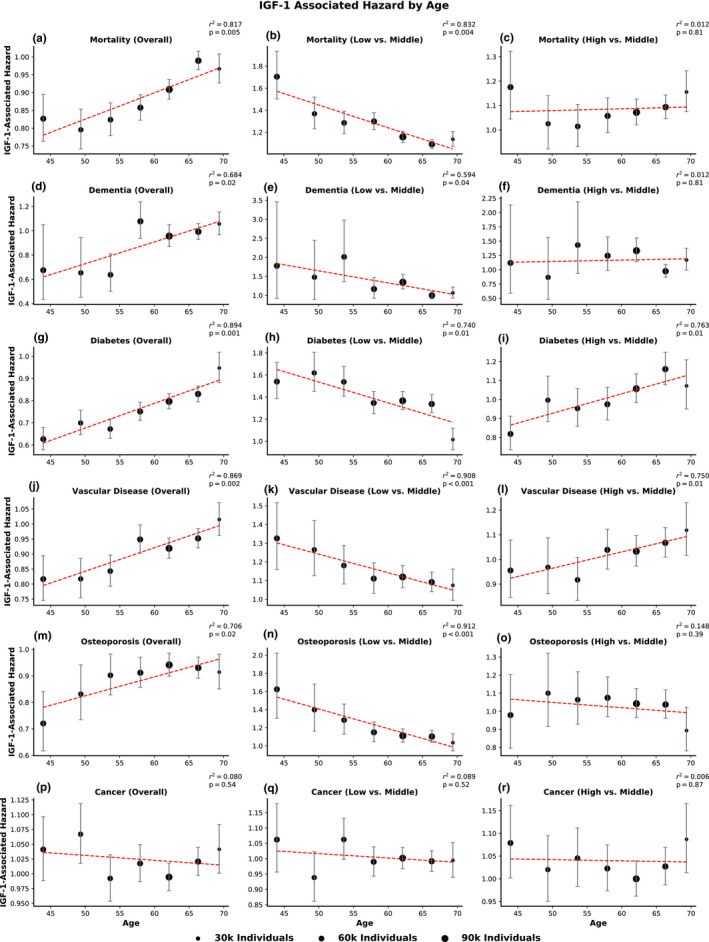
Clinical hazard associated with IGF‐1 increases with age, but not for cancer. In the left column, each dot represents the hazard ratio for incident disease or mortality associated with one standard deviation higher serum IGF‐1 *within* an age group, controlled for sex and residual age differences. In the middle and right columns, each dot represents the hazard ratio for incident disease or mortality associated with membership in the lowest or highest IGF‐1 quintiles, respectively, relative to the middle IGF‐1 quintile. For all subfigures, the area of each dot is proportional to the number of individuals it represents. Error bars indicate 95% confidence intervals for the hazard ratio. The reported *p*‐values are for an *F*‐test for linear regression among the groups, and the reported *r* ^2^ values are for a Pearson association between the linear fit line (dotted red line) and the hazard ratios. The clinical events evaluated were (a–c) mortality, (d–f) dementia, (g–i) diabetes, (j–l) vascular disease, (m–o) osteoporosis, and (p–r) cancer

Again, the clinical outcomes of mortality, dementia, diabetes, osteoporosis, and vascular disease all showed strikingly concordant results. When analyzed across the entire spectrum of IGF‐1 variation, IGF‐1 overall appears to be associated with protection against clinical events (Figure 3, left column). However, this protection is not uniform at all ages, but is more pronounced in the young. In younger individuals, higher IGF‐1 appeared to be associated with a lower hazard for clinical events. However, for older groups of individuals, IGF‐1 showed less protection from incident disease. For example, the hazard ratio for mortality associated with IGF‐1 is 0.83 (95% CI 0.76–0.90) for the youngest age group and 0.97 (95% CI 0.93–1.01) for the oldest age group. Moreover, significant positive correlations were noted between the hazard associated with high IGF‐1 and age for dementia (p=0.02), diabetes (p=0.001), vascular disease (p=0.002), osteoporosis (p=0.02), and mortality (p=0.005). Notably, cancer exhibited unique behavior compared to the other conditions; higher IGF‐1 was generally associated with increased hazard for incident cancer, with no significant trend across different ages (p=0.54). When controlled for body mass index (BMI), these effects all remained significant (Figure S6).

To explore sex‐specific effects, we conducted stratified analyses for men and women (Figures S7 and S8). The age‐interaction effect was significant across both sexes for diabetes (men $p<10^{‐4}$, women p=0.005), vascular disease (men p=0.003, women p=0.002), osteoporosis (men p=0.04, women p=0.04), and mortality (men p=0.008, women p=0.01). Trends for dementia reached significance in women (p=0.04) but not in men (p=0.06). As in the overall analysis, no significant age‐interaction effect was noted for cancer in either sex (men p=0.33; women p=0.69).

Although we identified an interaction between age and IGF‐1, these models do not account for the earlier finding of a U‐shape relationship between clinical outcomes and IGF‐1. To clarify the relationship between the highest and lowest IGF‐1 levels and clinical hazard, we conducted age‐interaction analyses explicitly focusing on the lowest (Figure 3, middle column) and highest (Figure 3, right column) quintiles of IGF‐1 when compared to the middle quintile. These analyses showed that the overall age‐interaction effect can be decomposed into two components: (a) The lowest IGF‐1, which is associated with the greatest hazard in younger individuals, becomes less harmful in older individuals (Figure 3, middle column); and (b) The highest IGF‐1 levels tend to be associated with relative protection in younger individuals, and become more harmful in older individuals (Figure 3, right column). These findings highlight the importance of considering both nonlinear relationships and age interactions in analyses of IGF‐1.

### 2.4 Age‐interaction and U‐shaped hazard represent independent effects

**FIGURE 4:**
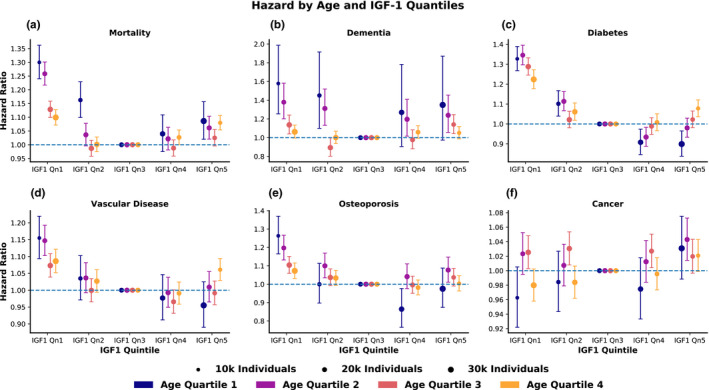
The U‐shape and age‐interaction effects are independent. After stratifying the population into sex‐specific age quartiles and IGF‐1 quintiles, each group's hazard relative to the age‐matched IGF‐1 quintile 3 is shown for (a) mortality, (b) dementia, (c) diabetes, (d) vascular disease, (e) osteoporosis, and (f) cancer. The area of each dot is proportional to the number of individuals it represents, and error bars indicate 95% confidence intervals for hazard ratios

To better illustrate the relationship between the U‐shape and age interaction effects, we stratified our cohort into 20 groups based on both IGF‐1 quintiles and age quartiles. Within each age quartile, we estimated the hazard ratio of each IGF‐1 quintile relative to the third IGF‐1 quintile, using Cox proportional hazards models (Figure 4 and Tables S1‐S6).

Overall, both the U‐shape and age‐interaction effects were present across age and IGF‐1 quantiles and diseases, though they were more prominent in the lower IGF‐1 quintiles. In general, the U‐shape effect persists within each age quartile; those in the highest and the lowest IGF‐1 quintiles usually have higher hazard than those in the third quintile. However, the size of the effect, which was most visually pronounced for mortality hazard, varied greatly. In particular, individuals in the lowest IGF‐1 quintiles tended to be at highest risk compared to those in the other IGF‐1 quintiles, showing that the U‐shape effect is not fully symmetric. Further, within the first IGF‐1 quintile, the hazard associated with IGF‐1 decreased over successively older age quartiles, demonstrating the age‐interaction effect for low IGF‐1. Conversely, the mirrored effect for high IGF‐1 was most prominent in IGF‐1 quintile 5 for diabetes and vascular disease: Within IGF‐1 quintile 5, younger individuals had the lowest hazard for incident disease, with risk increasing for older age groups. While these effects differ in specific shape and relative strength across diseases, they broadly hold across most clinical outcomes, except for cancer. Sex stratification and controlling for BMI yielded largely similar results (Figures S10, S11, and S9).

**FIGURE 5:**
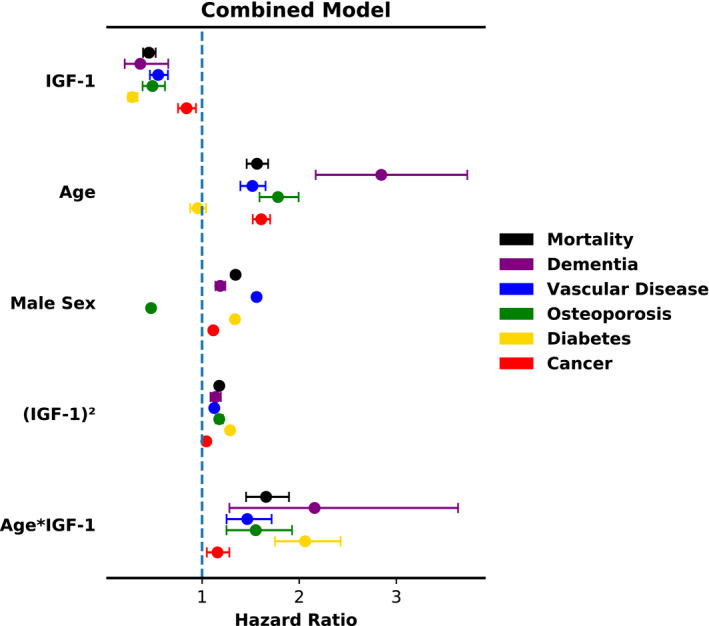
The U‐shape and age‐interaction effects are consistent across varied conditions in a combined Cox model. The (IGF‐1)^2^ term represents the U‐shape effect, while the Age*IGF‐1 term represents the age‐interaction effect. A linear IGF‐1 term, age, and sex are also included as covariates

Lastly, to more rigorously test the interactions between age and IGF‐1 without manual selection of stratification groups, we built a single combined model with all the individuals in our cohort (Figure 5 and Table S7). In addition to a linear IGF‐1 term, age, and sex, we included (IGF‐1)^2^ and Age*IGF‐1 interaction terms in this combined model to assess the overall significance of the U‐shape and age‐IGF‐1 interaction effects, respectively. The (IGF‐1)^2^ term generally measures the extent to which extreme values of IGF‐1, both high and low, are associated with adverse outcomes. Further, the Age*IGF‐1 interaction term measures the extent to which the hazard associated with IGF‐1 increases with age; it is conceptually related to the slope of the fit lines in Figure 3. Consistent with our stratified analyses, we found the directions of clinical event hazards associated with these terms to be highly significant and consistent in direction for both the age‐interaction (mortality $p<10^{‐13}$; dementia p=0.004; vascular disease $p<10^{‐5}$; osteoporosis p<0.001; diabetes $p<10^{‐17}$) and U‐shape effect terms (mortality $p<10^{‐96}$; dementia $p<10^{‐7}$; vascular disease $p<10^{‐17}$; osteoporosis $p<10^{‐23}$; diabetes $p<10^{‐165}$). Interestingly, the combined analysis also showed weaker but still significant effects for cancer as well (age‐interaction p=0.004; U‐shape p=0.001).

The results were also similar when stratified by sex (Figures S13 and S14), with some associations becoming only nominally significant. In women, the age‐interaction term for dementia and both the (IGF‐1)^2^ term and age‐interaction term for cancer become no longer statistically significant, while all associations remained at least nominally significant in men. All reported associations for both the U‐shape (IGF‐1)^2^ term and the age‐interaction term remained significant when controlled for BMI (Figure S12).

## 3 DISCUSSION

In this study, we have shown that the relationship between IGF‐1 and age‐associated disease is generalized across a variety of disease states with distinct physiology. Using the power of the hundreds of thousands of participants in the UK Biobank, we have resolved an important controversy on the relationship of IGF‐1 and disease risk. Further, we have uncovered a unifying framework by which the wide range of results in previous studies can be understood: IGF‐1 is a nonlinear predictor of risk and interacts with age to modify risk for a variety of clinical events. This extends our knowledge of the insulin/IGF‐1 pathway, an important modulator of aging in model organisms, to humans, and further provides direct evidence for antagonistic pleiotropy, an evolutionary theory for the origin of aging itself.

While previous studies focused on IGF‐1's age‐independent effect, we have discovered a novel interaction between IGF‐1 and age: While IGF‐1 is generally associated with protection from disease in younger individuals, it is conversely associated with increased risk for morbidity in older individuals. As a key player in the growth hormone pathway, IGF‐1 is central to the regulation of growth and development, important processes during youth. Conversely, it may inhibit core processes of repair and maintenance for older individuals, becoming harmful in old age. This is consistent with evidence from human cohorts with exceptional longevity, which have been shown to be enriched in variants in genes in the IGF‐1 pathway, including IGF1R, AKT1, and FOXO3A (Pawlikowska et al., 2009). In particular, specific variants in IGF1R have been shown to confer partial IGF‐1 resistance (Suh et al., 2008), providing functional evidence that downregulation of the IGF‐1 pathway may prolong lifespan.

One mechanism by which IGF‐1 may be detrimental to older adults is through the inhibition of autophagy (Troncoso et al., 2012), a critical maintenance process particularly in older individuals. In model organisms and *in vitro* studies, autophagy has been shown to mediate protective effects on a wide variety of organ systems (Rubinsztein et al., 2011), including glucose metabolism, the heart, bone, and the nervous system. In addition, IGF‐1 signaling is also known to promote downstream mTOR signaling (Taniguchi et al., 2006), which may decrease lifespan (Papadopoli et al., 2019) and further inhibit autophagy through mTOR‐dependent mechanisms (Al‐Bari & Pingyong, 2020). While IGF‐1 as a whole is critical to the growth and development of humans, its inhibition of autophagy may contribute to its increasing association with morbidity and mortality with age.

Furthermore, when IGF‐1 signaling was experimentally attenuated in a group of older mice, improvements were observed in female lifespan and healthspan. In a recent study, a monoclonal antibody against the IGF‐1 receptor, IGF‐1R, was used to treat a group of 18‐month old mice, thereby decreasing downstream IGF‐1 signaling. In females, median lifespan was significantly increased, while inflammation and neoplasms were decreased (Mao et al., 2018). Taken together, evidence from centenarian genetics, cell biology, preclinical interventional trials, and now biodemography all support a potentially adverse role for IGF‐1 in human aging, particularly in older individuals. This is in stark contrast to its crucial and likely disease‐protective role in the growth and development of younger individuals. Thus, serum IGF‐1 may be a key example of antagonistic pleiotropy in humans, and, to the best of our knowledge, the first to be explicitly observed in relation to *bona fide* clinical events.

While most age‐related conditions exhibited complex U‐shaped and age‐interaction effects, these effects were substantially weaker for cancer. When higher‐order interaction effects are not accounted for, higher IGF‐1 appeared to be more consistently associated with cancer risk across ages. This may be due to the fact that IGF‐1 is directly involved in cancer pathogenesis. It is well established that IGF‐1 promotes cellular growth and proliferation through critical signaling pathways such as the IRS/Akt/MAPK pathway (Novosyadlyy & LeRoith, 2012). Thus, any indirect effect that IGF‐1 may have on cancer risk through modulating a global aging pathway might be overshadowed by its direct, cancer‐promoting effects.

In this study, we have also extended previous work on the U‐shaped relationship between IGF‐1 and disease risk. While this association has been shown in prior studies (Andreassen et al., 2009; Burgers et al., 2011; Svensson et al., 2012), we have demonstrated that it is more general than previously appreciated. Strikingly, it seems to apply to the majority of clinically relevant age‐associated diseases. In concert with the previously discussed interaction between age and IGF‐1, this suggests that IGF‐1 may be modulating a common underlying pathway for many age‐associated conditions and, potentially, aging itself.

While high IGF‐1 may be causally associated with accelerated aging and increased age‐associated pathology, the mechanistic connection between low IGF‐1 and clinical risk is not well‐understood. One possible explanation stems from the fact that IGF‐1 is lower in older individuals; high IGF‐1 may therefore be considered a biomarker for youth. As a result, it is possible that some individuals with low IGF‐1 currently may have actually experienced accelerated aging—potentially even due to, paradoxically, previously elevated levels of IGF‐1. Further, low IGF‐1 is known to occur in patients with either chronic (Juul, 2003) or acute illness (Ross et al., 1991). Therefore, it is possible that some individuals have low IGF‐1 due to underlying latent disease processes. Thus, low IGF‐1 may be caused by undetected pre‐existing disease, rather than low IGF‐1 itself causing future disease.

One limitation is the lack of longitudinal serum IGF‐1 measurements over time. While a clear decline in IGF‐1 levels in older adults is observed cross‐sectionally, much remains unknown about the longitudinal dynamics of IGF‐1 levels in individuals. It is unclear (a) how stable IGF‐1 levels are for individuals over time; (b) whether IGF‐1 self‐inhibits over time through accelerated aging; and (c) whether trajectories of IGF‐1 levels are predictive of distinct clinical outcomes. While the UK Biobank provides detailed longitudinal follow‐up for survival events, serum IGF‐1 measurements were only available at baseline for the majority of participants. Longitudinal studies with repeated measurements of IGF‐1 are needed to further explore this question.

Other approaches, such as Mendelian randomization, may also help to clarify the directionality of causality in these associations. One Mendelian randomization study in the UK Biobank found associations between higher genetically determined IGF‐1 and diabetes and vascular disease (Larsson et al., 2020), consistent with our results. However, a different study in the same cohort failed to find any significant associations between genetically determined IGF‐1 and 40 medically relevant phenotypes (Sinnott‐Armstrong et al., 2021). In addition, it has been established that obesity is associated with higher free IGF‐1 levels (Nam et al., 1997), and there is evidence from the Long Life Family Study of a nonlinear relationship between IGF‐1 levels and BMI (Sherlala et al., 2021). We therefore performed supplemental analyses controlled for BMI and found that our results were not substantially altered. While it remains possible that BMI may partially mediate some of the complex effects we observed, they appear to be largely independent of the effect of BMI. Additional work is required to further clarify the causal relationship between IGF‐1 and disease.

Utilizing the largest IGF‐1 dataset available to date, we demonstrated a U‐shaped relationship between serum IGF‐1 levels and most major diseases and mortality. Furthermore, the findings exhibited a clear interaction between age and IGF‐1 level, which we have shown to strongly influence the role of IGF‐1 in disease risk. More generally, the strong consistency of hazard associations for multiple diseases suggests that the morbidities of old age are intricately interrelated and intertwined, which may be explained by the existence of an underlying unified aging process. In fact, these results suggest that IGF‐1 may be one of the central regulators of that overarching aging process in humans, promoting increased growth and anabolism at the cost of autophagy and somatic maintenance.

## 4 EXPERIMENTAL PROCEDURES

### 4.1 Study cohort

The UK Biobank is a population‐based study of roughly 500,000 individuals from the United Kingdom (Bycroft et al., 2018). It provides baseline measurements at recruitment between 2006 and 2010, and follows participants prospectively through integration with national health records. At baseline, participants underwent extensive phenotyping, including phlebotomy and physical measurements, and provided medical history and demographic information. Due to relatively lower numbers of other ethnicities in the UK Biobank, this study focused on individuals of European descent. We included 440,185 individuals of self‐reported European ancestry for whom serum insulin‐like growth factor‐1 (IGF‐1) measurements were available. This study was approved by the institutional review board (IRB) at the Albert Einstein College of Medicine. Informed consent was obtained from all study participants by the UK Biobank.

#### 4.1.1 IGF‐1 measurements

IGF‐1 measurements were made by chemiluminescence on a Liaison XL (Diasorin Ltd., Saluggia, Italy). The limit of quantification (LOQ) was 1.30 nmol/L and the coefficient of variance (CV) was 6.03%, 5.29%, and 6.18% for the low (range 7.06–12.84 nmol/L), medium (range 27.23–44.97 nmol/L), and high (range 35.85–84.62 nmol/L) internal quality controls, respectively. More detailed information about UK Biobank biochemical measurements can be found online (https://biobank.ctsu.ox.ac.uk/crystal/crystal/docs/serum_biochemistry.pdf).

#### 4.1.2 Clinical event definitions: Mortality

Mortality was assessed using the UK Biobank's data from linkage to national death registries. Follow‐up occurred through April 2020 for mortality and morbidities listed as causes of death (death register), through March 2020 (England), October 2016 (Scotland), and February 2016 (Wales) for morbidities as defined by inpatient diagnosis codes, and through March 2016 (England and Wales) and October 2015 (Scotland) for cancer diagnoses (cancer register).

#### 4.1.3 Pre‐defined clinical events: Dementia and vascular disease

Dementia was assessed using the UK Biobank's internal algorithmically defined dementia variable. This variable is based on a combination of inpatient and death register ICD‐10 and ICD‐9 codes, and self‐report, as previously described (Wilkinson et al., 2018). A detailed description of the procedure and a full list of ICD codes used in the definition of this variable is available online (https://biobank.ctsu.ox.ac.uk/crystal/crystal/docs/alg_outcome_dementia.pdf).

Vascular disease was defined as a composite of stroke and myocardial infarction (MI). Stroke and myocardial infarction were assessed using internal algorithmically defined variables from the UK Biobank. As with the dementia variable, these variables were built using a combination of ICD‐10 codes, ICD‐9 codes, and self‐report, with a full description available on the UK Biobank's website (stroke: https://biobank.ctsu.ox.ac.uk/crystal/crystal/docs/alg_outcome_stroke.pdf; MI: https://biobank.ctsu.ox.ac.uk/crystal/crystal/docs/alg_outcome_mi.pdf). For this study, prevalent vascular disease was defined as the occurrence of either MI or stroke prior to the baseline assessment. Incident vascular disease was defined as the first occurrence of either MI or stroke following the baseline assessment, excluding individuals with prevalent vascular disease.

#### 4.1.4 ICD‐defined clinical events: Diabetes, osteoporosis, and cancer

Diabetes was not available as an algorithmically defined variable from the UK biobank and was therefore constructed using a similar approach for this study. The earliest occurrence of either an inpatient ICD‐10 code in the range E10–E14, an inpatient ICD‐9 code in the range 250, a death register entry with an ICD‐10 or ICD‐9 code in either range, or a self‐report from UK Biobank data‐field 2976 was considered to be the date of disease onset. When the death register entry was the earliest documentation of disease, the date of death was considered to be the date of onset. Prevalent diabetes was defined as a date of disease onset before the baseline assessment. Incident diabetes was defined as a date of disease onset following the baseline assessment, excluding individuals with prevalent diabetes.

Osteoporosis was defined as the earliest occurrence of either ICD‐10 code in the range M80–M82, or ICD‐9 code 7330, using inpatient and death register data. As with diabetes, prevalent osteoporosis was defined as a date of disease onset before the baseline assessment, and when a death register entry was the earliest documentation of disease, the date of death was considered to be the date of onset. Incident osteoporosis was defined as a date of disease onset following the baseline assessment, excluding individuals with prevalent osteoporosis.

Cancer was defined as the earliest occurrence of a malignant neoplasm, excluding non‐melanoma cancers of the skin. The UK Biobank used national cancer registries to obtain follow‐up data on cancer in its participants.

For this study, the occurrences of ICD‐10 codes C00–C97, excluding C44, or ICD‐9 codes 140–209, excluding 173, in the cancer registry were considered as cancer diagnoses. Prevalent and incident cancer were defined as a date of disease onset prior to or following the baseline assessment, respectively. Incident cancer analyses excluded individuals with prevalent cancer. A full listing of ICD codes and UK Biobank self‐report fields used for diabetes, osteoporosis, and cancer is provided in Table S8.

### 4.2 Stratification by age and IGF‐1

For the separate age‐interaction (Figure 3) and U‐shape (Figure 2) analyses, the cohort was split into groups based on the distribution of age and IGF‐1 ranges, respectively. After excluding individuals with baseline disease for each morbidity, a total of seven groups were created based on evenly spaced intervals between the 5th percentile and 95th percentile value of age or IGF‐1. The individuals in the top and bottom 5% were then appended to the highest (group 7) and the lowest (group 1) groups, respectively. This procedure produced evenly spaced intervals with relatively consistent numbers of individuals within each group (since outliers at each extreme are grouped together). This nonparametric approach has the advantage of being able to capture nonlinear relationships between IGF‐1 and risk of disease, which may be overlooked when IGF‐1 is used as a parameter in a linear model.

### 4.3 Statistical analysis

Statistical analysis was performed using custom scripts in Python (version 3.6), including standard numeric and scientific packages such as numpy, pandas, and scipy. Cox proportional hazard models were fit using the package lifelines (Davidson‐Pilon et al., 2019). All covariates in Cox analyses were rescaled to a mean of 0 and standard deviation of 1.

#### 4.3.1 Age‐interaction analysis

In the overall age‐interaction analysis (Figure 3, left column), Cox proportional hazards models were fit to determine the hazard associated with one standard deviation increase in IGF‐1 *within* each age group. Age and sex were included as covariates. In the age‐interaction analyses focused on the lowest (Figure 3, middle column) and highest (Figure 3, right column) quintiles of IGF‐1, Cox proportional hazards models were fit to determine the hazard associated with membership in that sex‐specific quintile of IGF‐1 relative to membership in the middle IGF‐1 quintile (e.g., Quintile 1 vs. Quintile 3). IGF‐1 group membership (in which membership in the focal quintile was coded as 1 and membership in the middle quintile as 0), age, and sex were included as covariates. For all of these analyses, an *F*‐test for linear regression was performed to assess the relationship between age and IGF‐1‐associated hazards.

#### 4.3.2 U‐shape analysis

In the U‐shape analysis (Figure 2), Cox proportional hazards models were fit to determine the relative hazard of experiencing a clinical event for each IGF‐1 range group. The middle group (group 4) was designated as the reference group for comparisons. For each non‐reference group, a Cox model with group membership, age, and sex as covariates was fit, and the estimated hazard ratio for each group relative to the reference group was obtained.

Due to the clearly nonlinear relationship between clinical event hazard and IGF‐1 levels, a quadratic model was fit to the data points for each clinical outcome. To confirm this nonlinear relationship, the residuals of a linear fit to the data were computed. A second quadratic model was then fit to those residuals and an *F*‐test for linear regression was performed between the second quadratic model and the residuals.

#### 4.3.3 IGF‐1 quintile and age quartile analysis

In order to understand the relationship between the age‐interaction and U‐shape effects, the cohort was simultaneously split into sex‐specific age quartiles (Age Q1‐4) and sex‐specific IGF‐1 quintiles (IGF‐1 Qn 1‐5) for a total of 20 groups of interest (Figure 4). For example, all subjects who are both in the sex‐specific fourth quartile of age (Age Q4), and in the sex‐specific first quintile of IGF‐1 (IGF‐1 Qn1) comprise one of these 20 groups. Then, the third IGF‐1 quintile (Qn3) was designated as the reference group for each age quartile. For each IGF‐1 quintile and age quartile, a Cox model was fit with group membership, age, and sex as covariates. Each of the non‐reference groups was then compared to the third IGF‐1 quintile within the same age quartile.

#### 4.3.4 Combined hazards model for overall cohort

Lastly, to assess the age‐interaction and U‐shape effects in the entire cohort, we formed a combined model with explicit terms for these effects. For the age‐interaction effect, we included an interaction term that multiplied IGF‐1 serum level by age. For the U‐shape effect, we included an IGF‐1‐squared term that multiplied serum IGF‐1 level by itself. For each outcome of interest, these two terms were then included as covariates along with age, sex, and a linear IGF‐1 term in a combined Cox proportional hazards model (Figure 5). The *p*‐values for the two key IGF‐1‐squared and age*IGF‐1 terms all remained significant when multiplied by a Bonferroni correction factor of 12 (2 key effects × 6 clinical conditions). An additional analysis in which these more complex terms were iteratively added to a “base” Cox model of linear IGF‐1, sex, and age showed that these effects are significant regardless of the order in which terms are added (Table S9).

## CONFLICTS OF INTEREST

The authors declare that they have no conflicts of interest.

## AUTHOR CONTRIBUTIONS

W.B.Z. and S.M. conceptualized the study. W.B.Z. designed the experiments, carried out the analysis, and wrote the first draft of the manuscript. S.M. and N.B. provided funding and resources for the study. W.B.Z., S.M., K.Y., and N.B. wrote subsequent drafts of the manuscript.

## Supporting information

Supplementary Material

## Data Availability

The UK Biobank is open to the global research community and data access can be applied for at https://www.ukbiobank.ac.uk/ (Bycroft et al., 2018).
