# Supplementary material for: The antagonistic pleiotropy of insulin‐like growth factor 1
Source: Aging Cell. 2021 Aug 7;20(9):e13443. doi: 10.1111/acel.13443 (PMC8441393; doi:10.1111/acel.13443)
Supplement: Supplementary file 1 — Supplementary Material [file ACEL-20-e13443-s001.pdf]

12. Supporting Information

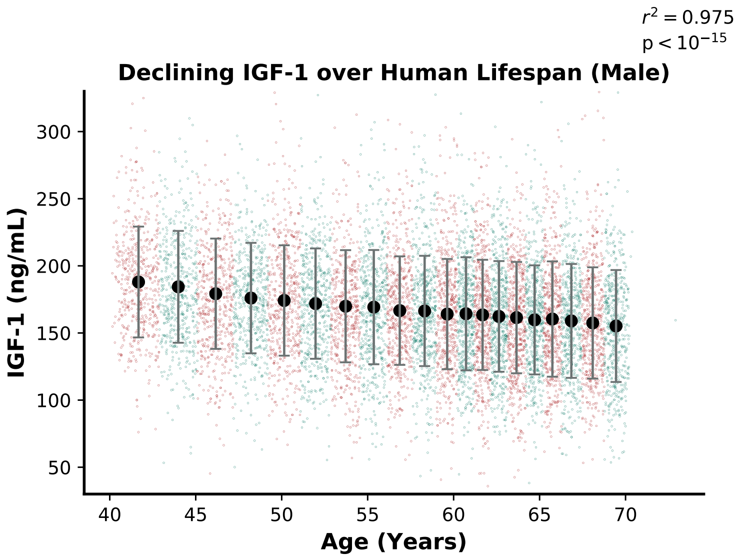

Figure S1: IGF-1 Distribution by Age, Males.

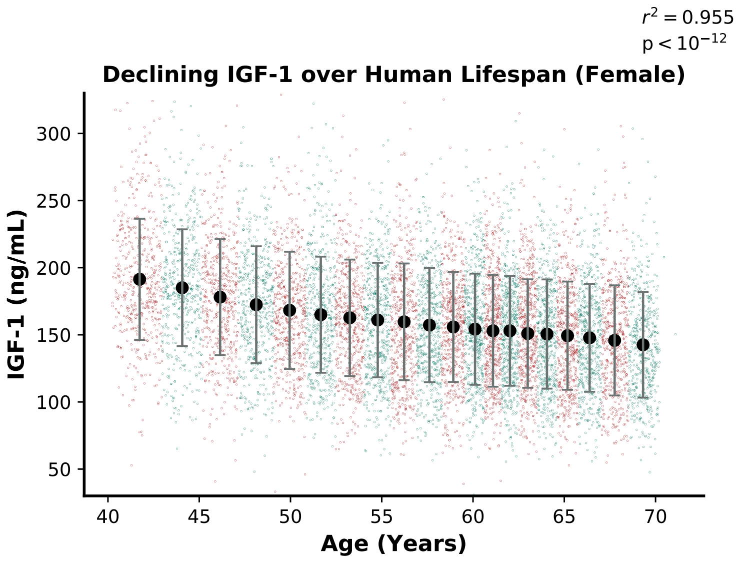

Figure S2: IGF-1 Distribution by Age, Females.

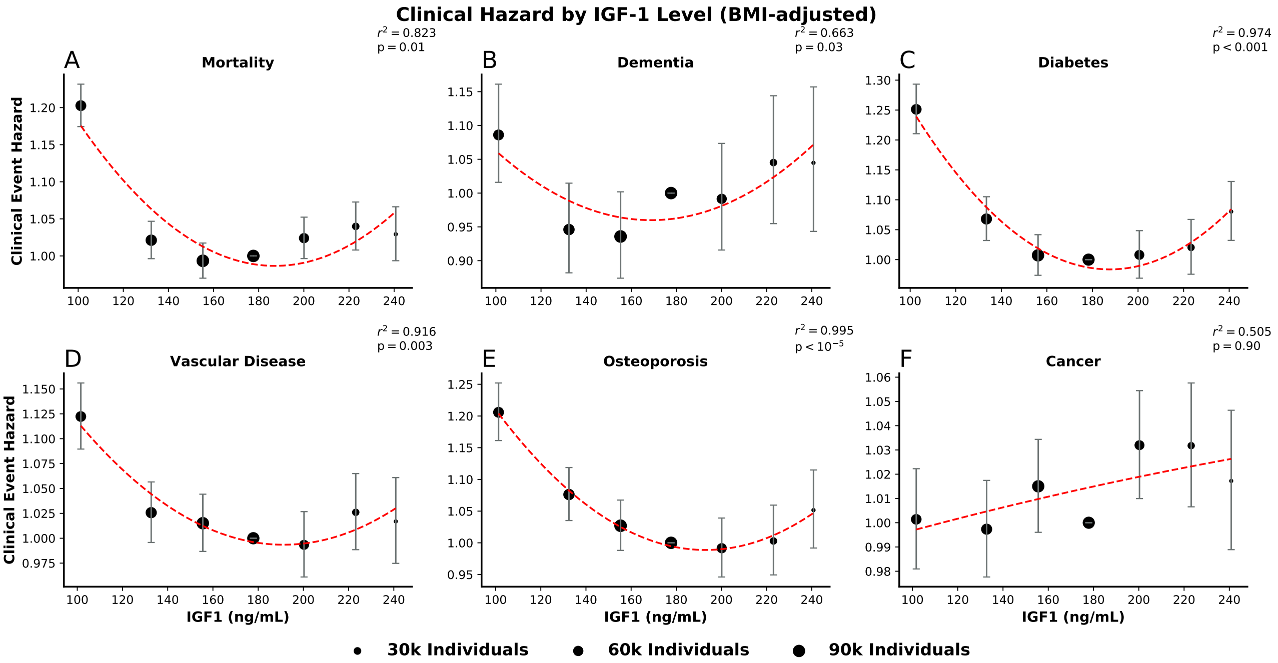

Figure S3: Hazard by IGF-1 Level, Controlled for BMI. The clinical events evaluated were (A) mortality, (B) dementia, (C) diabetes, (D) vascular disease, (E) osteoporosis, and (F) cancer.

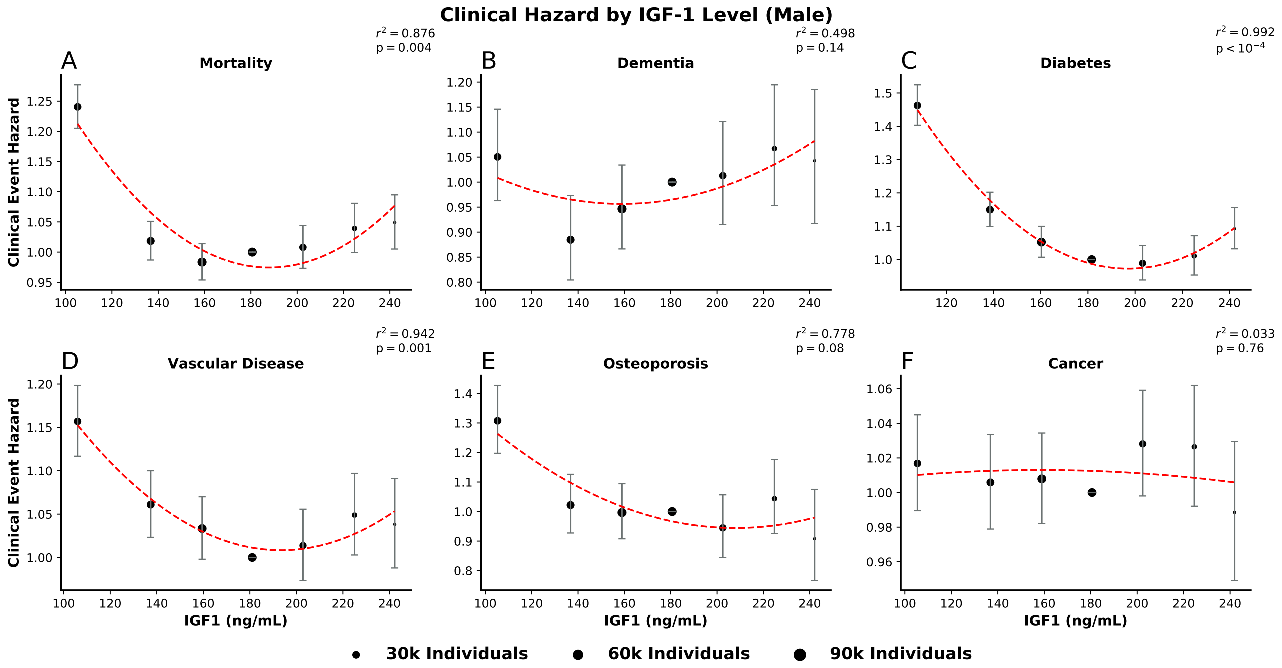

Figure S4: Hazard by IGF-1 Level, Males. The clinical events evaluated were (A) mortality, (B) dementia, (C) diabetes, (D) vascular disease, (E) osteoporosis, and (F) cancer.

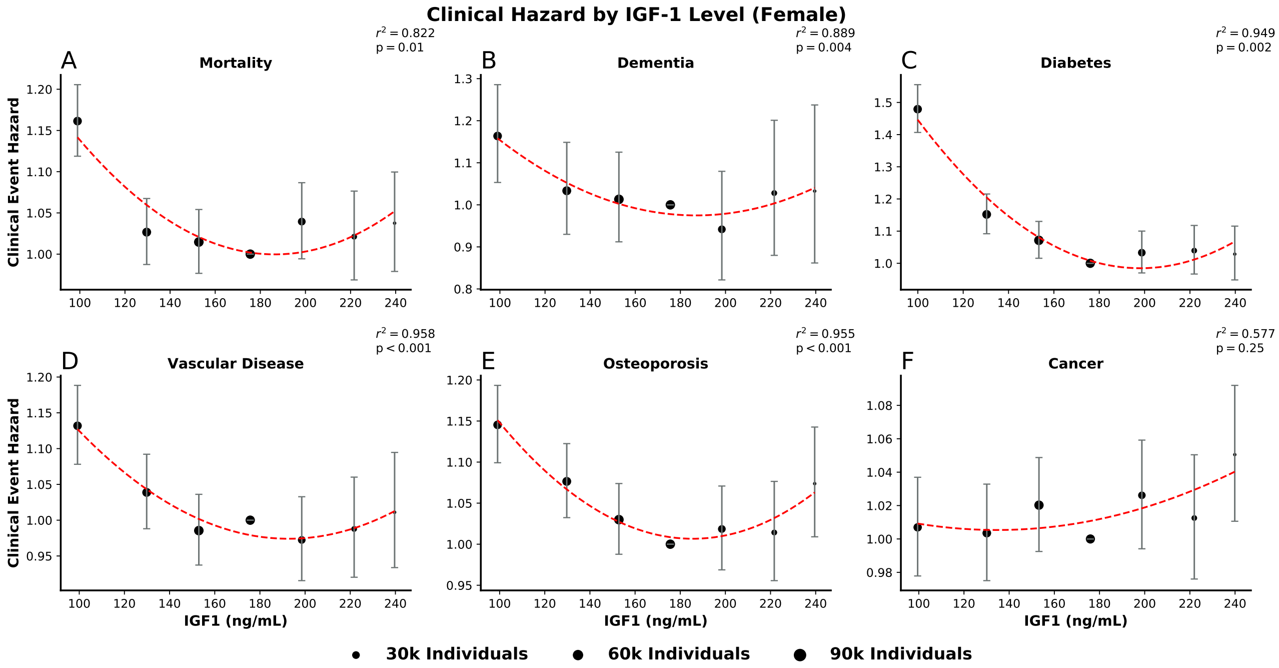

Figure S5: Hazard by IGF-1 Level, Females. The clinical events evaluated were (A) mortality, (B) dementia, (C) diabetes, (D) vascular disease, (E) osteoporosis, and (F) cancer.

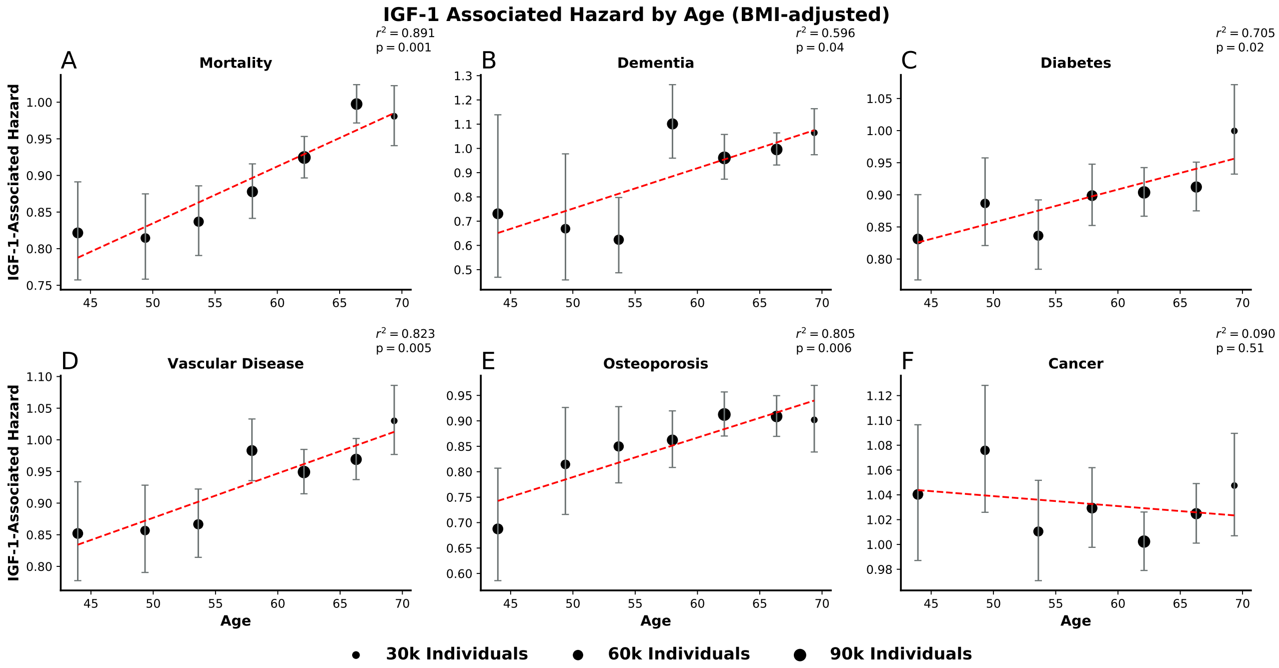

Figure S6: IGF-1-Associated Hazard by Age, Controlled for BMI. The clinical events evaluated were (A) mortality, (B) dementia, (C) diabetes, (D) vascular disease, (E) osteoporosis, and (F) cancer.

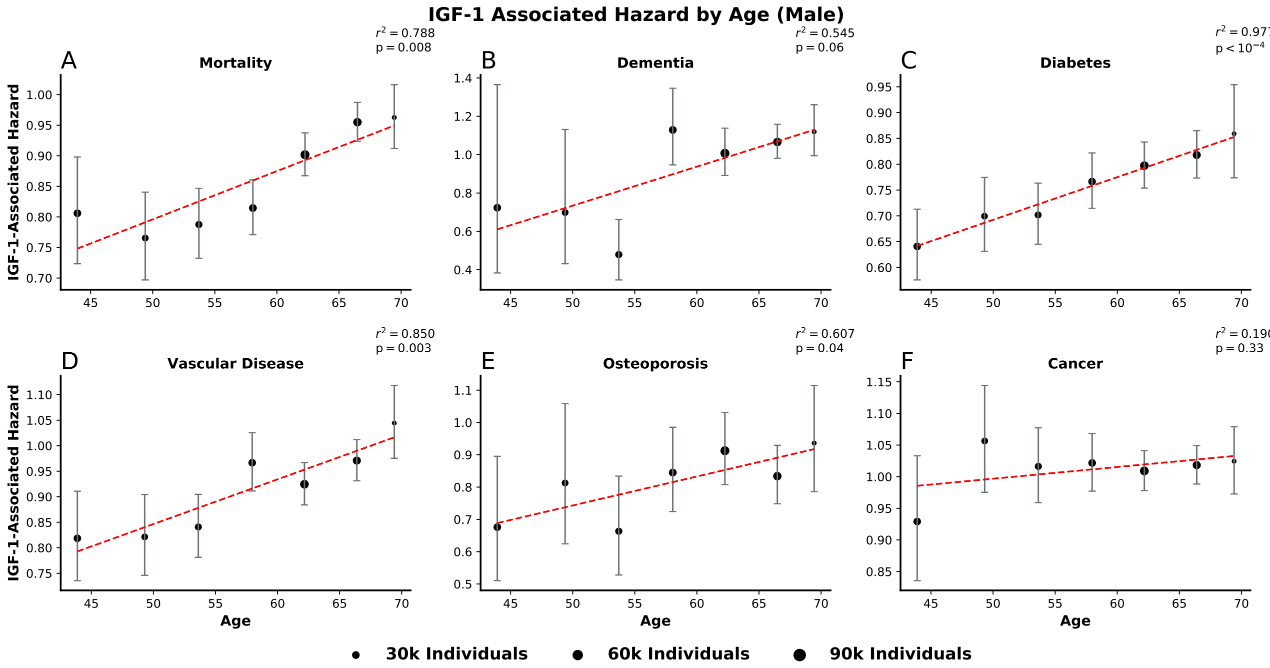

Figure S7: IGF-1-Associated Hazard by Age, Males. The clinical events evaluated were (A) mortality, (B) dementia, (C) diabetes, (D) vascular disease, (E) osteoporosis, and (F) cancer.

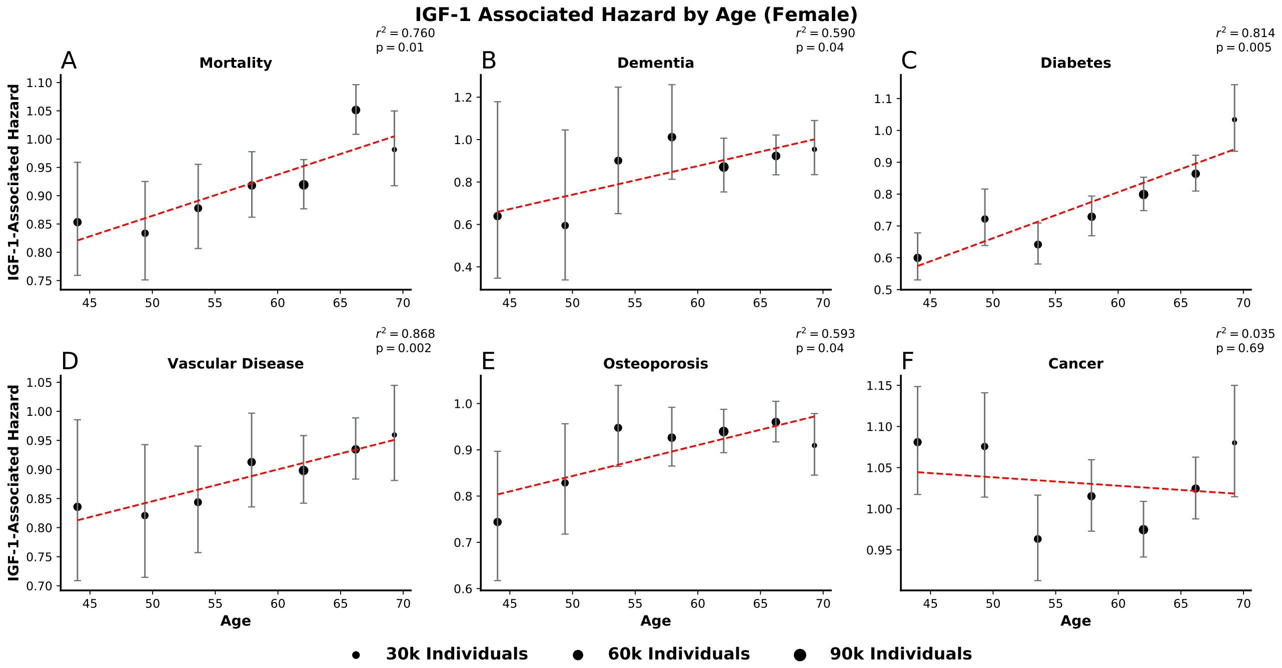

Figure S8: IGF-1-Associated Hazard by Age, Females. The clinical events evaluated were (A) mortality, (B) dementia, (C) diabetes, (D) vascular disease, (E) osteoporosis, and (F) cancer.

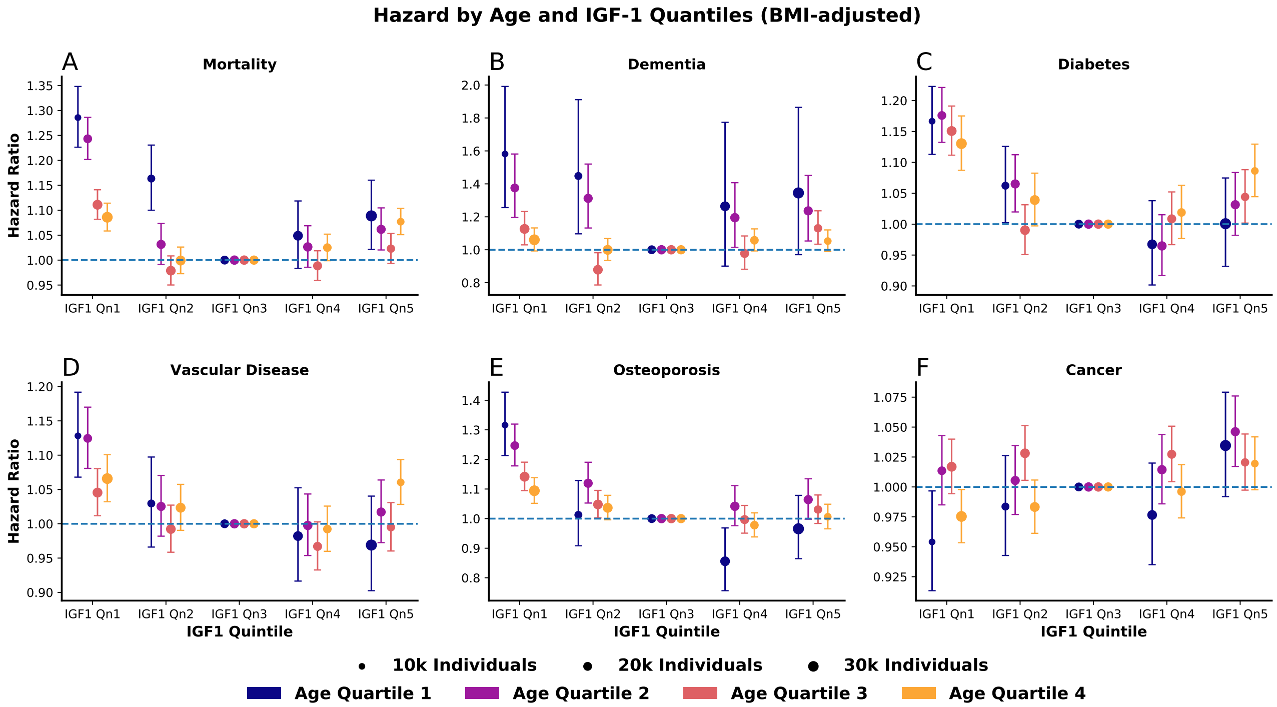

Figure S9: Mortality Hazard by Age and IGF-1 Quantiles, Controlled for BMI.

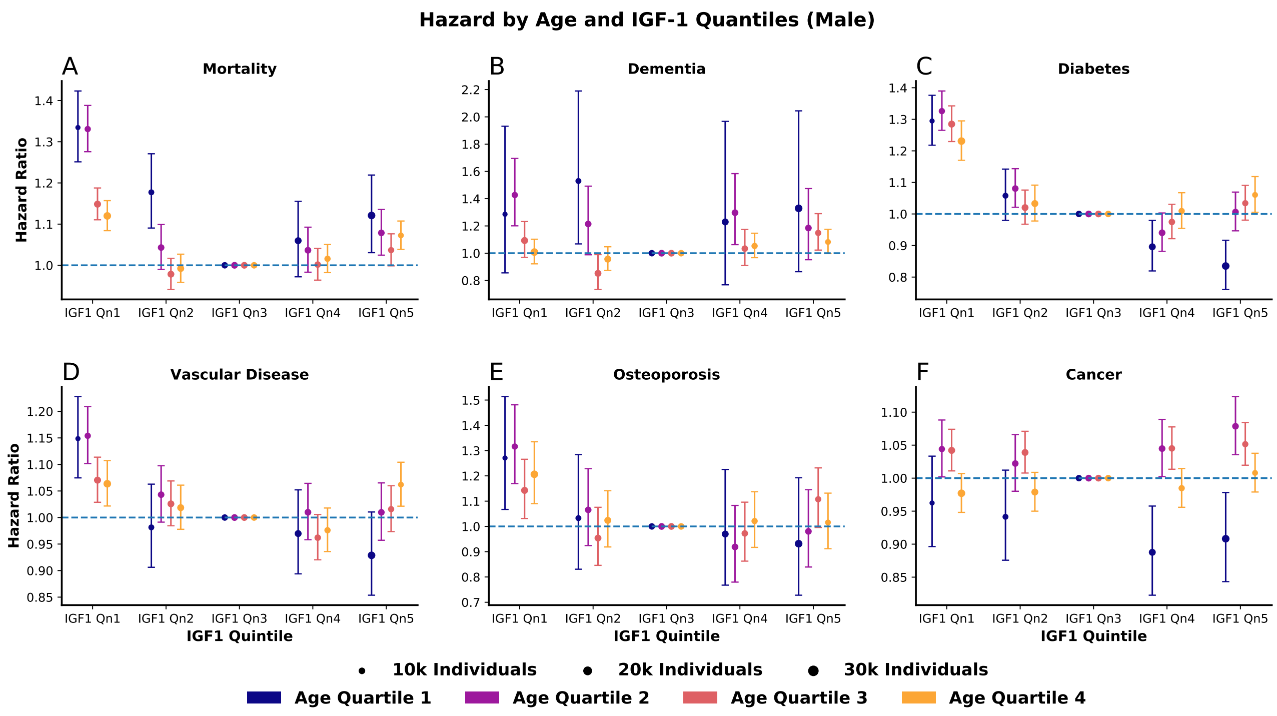

Figure S10: Mortality Hazard by Age and IGF-1 Quantiles, Males.

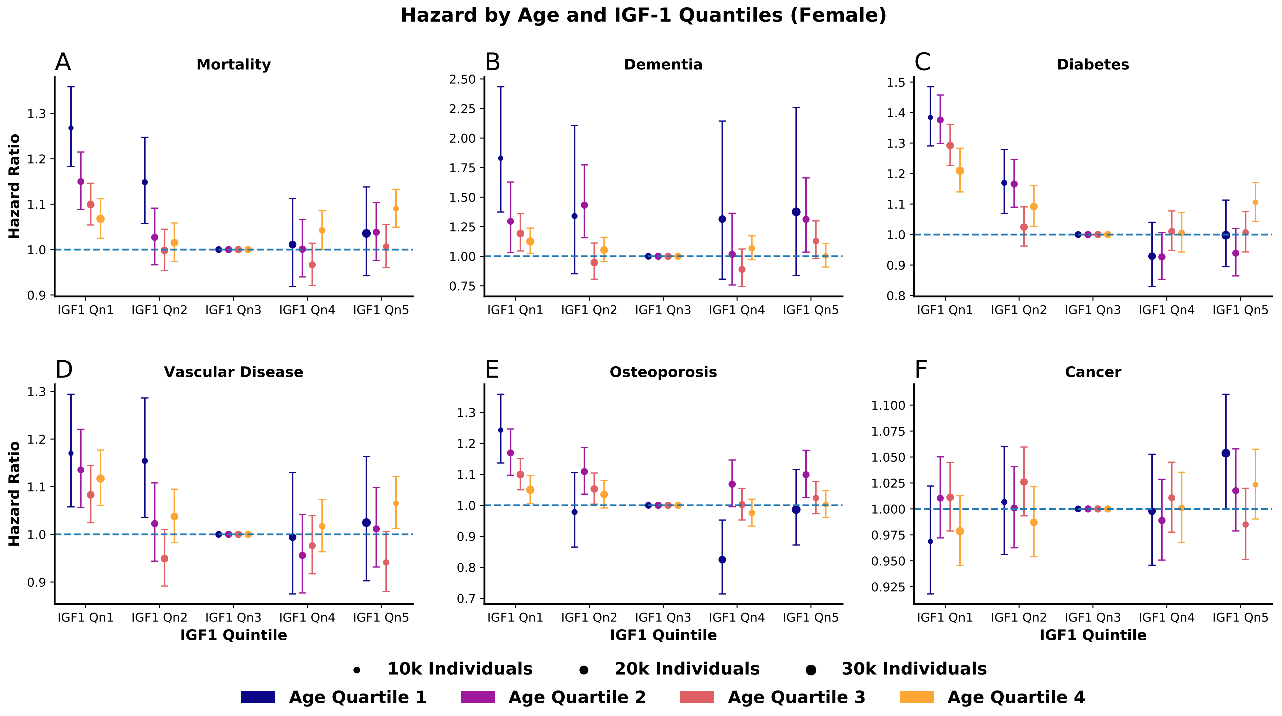

Figure S11: Mortality Hazard by Age and IGF-1 Quantiles, Females.

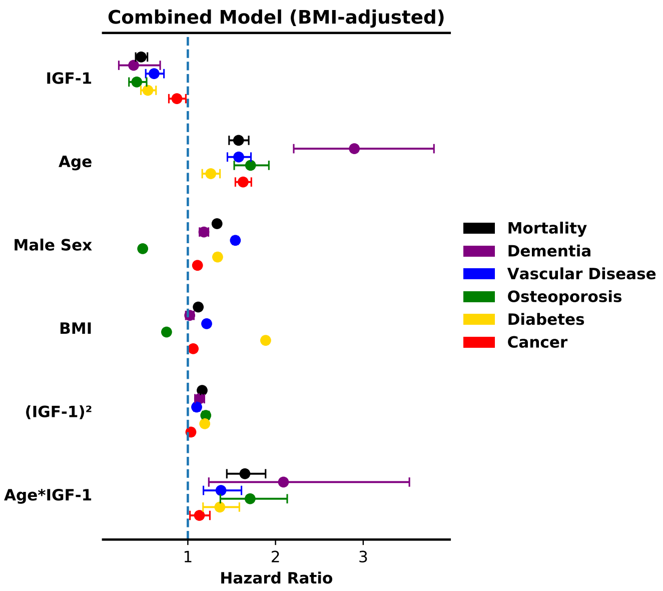

Figure S12: Hazards in Combined Cox Model, Controlled for BMI.

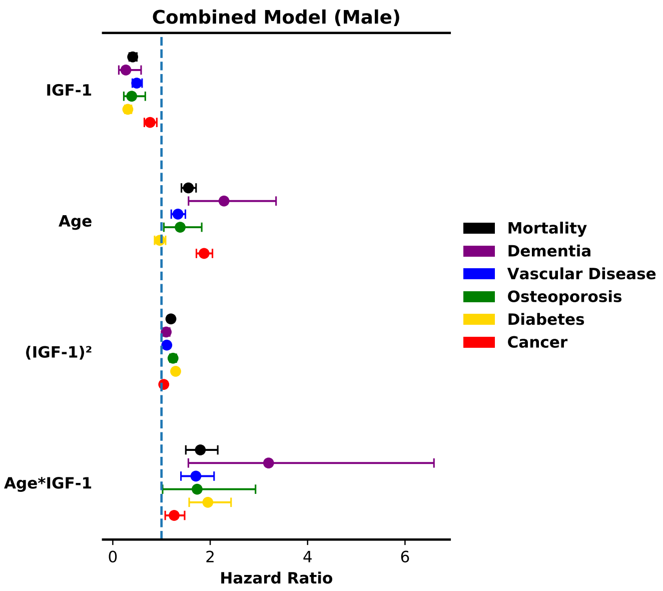

Figure S13: Hazards in Combined Cox Model, Males.

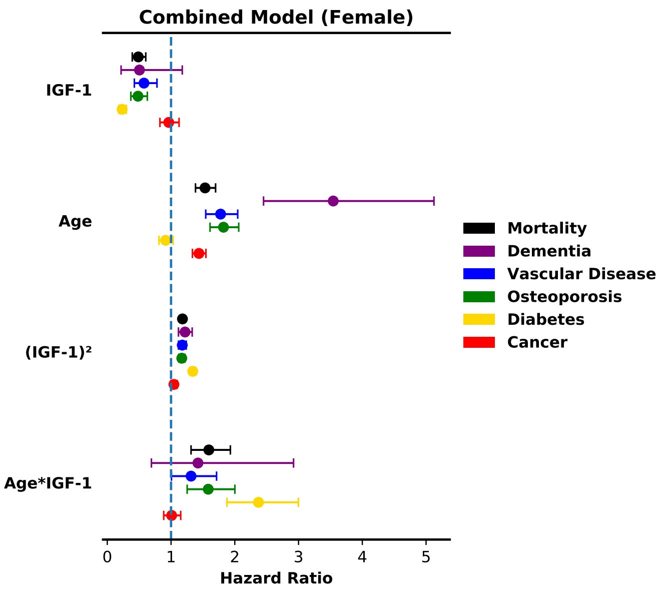

Figure S14: Hazards in Combined Cox Model, Females.

Mortality (HR (95% CI))				
	Age Q1	Age Q2	Age Q3	Age Q4
IGF1 Qn1	1.30 (1.24-1.36, $p < 10^{-27}$)	1.26 (1.22-1.30, $p < 10^{-40}$)	1.13 (1.10-1.16, $p < 10^{-18}$)	1.10 (1.07-1.13, $p < 10^{-12}$)
IGF1 Qn2	1.16 (1.10-1.23, $p < 10^{-6}$)	1.04 (1.00-1.08, $p = 0.08$)	0.99 (0.96-1.02, $p = 0.38$)	1.00 (0.97-1.03, $p = 0.93$)
IGF1 Qn3	1.00 (1.00-1.00, $p : N/A$)	1.00 (1.00-1.00, $p : N/A$)	1.00 (1.00-1.00, $p : N/A$)	1.00 (1.00-1.00, $p : N/A$)
IGF1 Qn4	1.04 (0.98-1.11, $p = 0.23$)	1.02 (0.98-1.06, $p = 0.30$)	0.99 (0.96-1.02, $p = 0.42$)	1.03 (1.00-1.05, $p = 0.04$)
IGF1 Qn5	1.09 (1.02-1.16, $p = 0.010$)	1.06 (1.02-1.10, $p = 0.003$)	1.03 (1.00-1.06, $p = 0.10$)	1.08 (1.05-1.11, $p < 10^{-9}$)

Table S1: Quantile hazards for mortality.

Dementia (HR (95% CI))				
	Age Q1	Age Q2	Age Q3	Age Q4
IGF1 Qn1	1.58 (1.25-1.99, $p < 0.001$)	1.38 (1.20-1.58, $p < 10^{-5}$)	1.14 (1.04-1.24, $p = 0.005$)	1.06 (1.00-1.13, $p = 0.07$)
IGF1 Qn2	1.45 (1.10-1.92, $p = 0.009$)	1.31 (1.13-1.52, $p < 0.001$)	0.89 (0.80-1.00, $p = 0.04$)	1.00 (0.94-1.07, $p = 0.98$)
IGF1 Qn3	1.00 (1.00-1.00, $p : N/A$)	1.00 (1.00-1.00, $p : N/A$)	1.00 (1.00-1.00, $p : N/A$)	1.00 (1.00-1.00, $p : N/A$)
IGF1 Qn4	1.27 (0.90-1.78, $p = 0.17$)	1.20 (1.02-1.41, $p = 0.03$)	0.98 (0.88-1.08, $p = 0.67$)	1.06 (0.99-1.13, $p = 0.08$)
IGF1 Qn5	1.35 (0.97-1.87, $p = 0.07$)	1.24 (1.06-1.45, $p = 0.009$)	1.14 (1.04-1.25, $p = 0.004$)	1.05 (0.99-1.12, $p = 0.12$)

Table S2: Quantile hazards for dementia.

Diabetes (HR (95% CI))				
	Age Q1	Age Q2	Age Q3	Age Q4
IGF1 Qn1	1.33 (1.27-1.39, $p < 10^{-33}$)	1.35 (1.30-1.40, $p < 10^{-56}$)	1.29 (1.25-1.33, $p < 10^{-48}$)	1.22 (1.18-1.27, $p < 10^{-23}$)
IGF1 Qn2	1.10 (1.04-1.17, $p = 0.001$)	1.11 (1.07-1.16, $p < 10^{-5}$)	1.02 (0.98-1.06, $p = 0.31$)	1.06 (1.02-1.11, $p = 0.005$)
IGF1 Qn3	1.00 (1.00-1.00, $p : N/A$)	1.00 (1.00-1.00, $p : N/A$)	1.00 (1.00-1.00, $p : N/A$)	1.00 (1.00-1.00, $p : N/A$)
IGF1 Qn4	0.91 (0.85-0.97, $p = 0.007$)	0.93 (0.89-0.98, $p = 0.009$)	0.99 (0.95-1.03, $p = 0.59$)	1.01 (0.97-1.05, $p = 0.72$)
IGF1 Qn5	0.90 (0.84-0.97, $p = 0.003$)	0.98 (0.93-1.03, $p = 0.42$)	1.02 (0.98-1.07, $p = 0.29$)	1.08 (1.04-1.12, $p < 0.001$)

Table S3: Quantile hazards for diabetes.

Vascular Disease (HR (95% CI))				
	Age Q1	Age Q2	Age Q3	Age Q4
IGF1 Qn1	1.15 (1.09-1.22, $p < 10^{-6}$)	1.15 (1.10-1.19, $p < 10^{-11}$)	1.07 (1.04-1.11, $p < 10^{-4}$)	1.09 (1.05-1.12, $p < 10^{-6}$)
IGF1 Qn2	1.03 (0.97-1.10, $p = 0.29$)	1.04 (0.99-1.08, $p = 0.10$)	1.00 (0.97-1.03, $p = 0.97$)	1.03 (0.99-1.06, $p = 0.11$)
IGF1 Qn3	1.00 (1.00-1.00, $p : N/A$)	1.00 (1.00-1.00, $p : N/A$)	1.00 (1.00-1.00, $p : N/A$)	1.00 (1.00-1.00, $p : N/A$)
IGF1 Qn4	0.98 (0.91-1.05, $p = 0.50$)	0.99 (0.95-1.04, $p = 0.75$)	0.97 (0.93-1.00, $p = 0.06$)	0.99 (0.96-1.02, $p = 0.59$)
IGF1 Qn5	0.96 (0.89-1.02, $p = 0.20$)	1.01 (0.97-1.06, $p = 0.69$)	0.99 (0.96-1.03, $p = 0.63$)	1.06 (1.03-1.09, $p < 0.001$)

Table S4: Quantile hazards for vascular disease.

Osteoporosis (HR (95% CI))				
	Age Q1	Age Q2	Age Q3	Age Q4
IGF1 Qn1	1.26 (1.16-1.37, $p < 10^{-7}$)	1.20 (1.13-1.27, $p < 10^{-9}$)	1.10 (1.06-1.15, $p < 10^{-5}$)	1.07 (1.03-1.12, $p < 0.001$)
IGF1 Qn2	1.00 (0.90-1.11, $p = 0.99$)	1.10 (1.03-1.17, $p = 0.002$)	1.04 (0.99-1.08, $p = 0.11$)	1.03 (0.99-1.08, $p = 0.10$)
IGF1 Qn3	1.00 (1.00-1.00, $p : N/A$)	1.00 (1.00-1.00, $p : N/A$)	1.00 (1.00-1.00, $p : N/A$)	1.00 (1.00-1.00, $p : N/A$)
IGF1 Qn4	0.86 (0.77-0.98, $p = 0.02$)	1.04 (0.98-1.11, $p = 0.22$)	1.00 (0.95-1.04, $p = 0.86$)	0.98 (0.94-1.02, $p = 0.38$)
IGF1 Qn5	0.98 (0.87-1.09, $p = 0.65$)	1.08 (1.01-1.15, $p = 0.02$)	1.04 (0.99-1.09, $p = 0.12$)	1.00 (0.96-1.05, $p = 0.86$)

Table S5: Quantile hazards for osteoporosis.

	Cancer (HR (95% CI))			
	Age Q1	Age Q2	Age Q3	Age Q4
IGF1 Qn1	0.96 (0.92-1.01, $p = 0.08$)	1.02 (0.99-1.05, $p = 0.11$)	1.03 (1.00-1.05, $p = 0.03$)	0.98 (0.96-1.00, $p = 0.08$)
IGF1 Qn2	0.98 (0.94-1.03, $p = 0.46$)	1.01 (0.98-1.04, $p = 0.62$)	1.03 (1.01-1.05, $p = 0.008$)	0.98 (0.96-1.01, $p = 0.16$)
IGF1 Qn3	1.00 (1.00-1.00, $p : N/A$)	1.00 (1.00-1.00, $p : N/A$)	1.00 (1.00-1.00, $p : N/A$)	1.00 (1.00-1.00, $p : N/A$)
IGF1 Qn4	0.97 (0.93-1.02, $p = 0.25$)	1.01 (0.98-1.04, $p = 0.40$)	1.03 (1.00-1.05, $p = 0.02$)	1.00 (0.97-1.02, $p = 0.69$)
IGF1 Qn5	1.03 (0.99-1.08, $p = 0.16$)	1.04 (1.01-1.07, $p = 0.003$)	1.02 (1.00-1.04, $p = 0.10$)	1.02 (1.00-1.04, $p = 0.06$)

Table S6: Quantile hazards for cancer.

Condition (HR (95% CI, p))					
	IGF-1	Age	Male Sex	$(\text{IGF-1})^2$	Age*IGF-1
Mortality	0.45 (0.39-0.52, $< 10^{-26}$)	1.57 (1.46-1.68, $< 10^{-34}$)	1.34 (1.32-1.37, $< 10^{-323}$)	1.18 (1.16-1.20, $< 10^{-96}$)	1.66 (1.45-1.89, $< 10^{-13}$)
Dementia	0.36 (0.20-0.65, < 0.001)	2.84 (2.17-3.73, $< 10^{-13}$)	1.19 (1.14-1.24, $< 10^{-14}$)	1.14 (1.09-1.19, $< 10^{-7}$)	2.16 (1.28-3.64, 0.004)
Vascular Disease	0.55 (0.46-0.65, $< 10^{-11}$)	1.52 (1.39-1.65, $< 10^{-21}$)	1.56 (1.53-1.59, $< 10^{-323}$)	1.13 (1.10-1.16, $< 10^{-17}$)	1.47 (1.25-1.72, $< 10^{-5}$)
Osteoporosis	0.49 (0.39-0.62, $< 10^{-8}$)	1.78 (1.59-1.99, $< 10^{-23}$)	0.48 (0.46-0.49, $< 10^{-323}$)	1.18 (1.14-1.21, $< 10^{-23}$)	1.55 (1.25-1.93, < 0.001)
Diabetes	0.28 (0.24-0.34, $< 10^{-48}$)	0.96 (0.88-1.04, 0.30)	1.34 (1.31-1.37, $< 10^{-172}$)	1.29 (1.27-1.31, $< 10^{-165}$)	2.06 (1.75-2.43, $< 10^{-17}$)
Cancer	0.84 (0.75-0.94, 0.002)	1.61 (1.52-1.70, $< 10^{-62}$)	1.12 (1.10-1.13, $< 10^{-68}$)	1.04 (1.02-1.07, 0.001)	1.16 (1.05-1.28, 0.004)

Table S7: Estimated hazards for each term in overall combined model. Corresponds to Figure 5.

See supplement_icd.tsv.

Table S8: ICD codes used for diabetes, osteoporosis, and cancer.

	+(IGF-1)^2	+Age*IGF-1	+Age*IGF-1	+(IGF-1)^2
Mortality	$p < 10^{-40}$	$p < 10^{-13}$	$p < 10^{-12}$	$p < 10^{-41}$
Dementia	$p < 0.001$	$p = 0.003$	$p = 0.004$	$p < 0.001$
Vascular Disease	$p < 10^{-8}$	$p < 10^{-5}$	$p < 10^{-5}$	$p < 10^{-9}$
Osteoporosis	$p < 10^{-9}$	$p < 10^{-4}$	$p < 0.001$	$p < 10^{-10}$
Diabetes	$p < 10^{-51}$	$p < 10^{-18}$	$p < 10^{-18}$	$p < 10^{-51}$
Cancer	$p = 0.009$	$p = 0.004$	$p = 0.008$	$p = 0.004$

Table S9: Significance of Adding Interaction Terms to Basic Cox Model. This analysis begins with a "base" Cox model consisting of a linear IGF-1 term, sex, and age. In the left half of the table, an (IGF-1)^2 term is added first, and then an Age*IGF-1 term, with significance being assessed after the inclusion of each additional term. Conversely, in the right half of the table, an Age*IGF-1 term is added first, and then an (IGF-1)^2 term.
